# A multistream attention based neural network for visual speech recognition and sign language understanding

**DOI:** 10.1038/s41598-025-26303-7

**Published:** 2025-12-24

**Authors:** Fatma M. Talaat, Basma M. Hassan

**Affiliations:** 1 Faculty of Artificial Intelligence, Kafrelsheikh University, Kafrelsheikh, 33516 Egypt; 2 Faculty of Computer Science & Engineering, New Mansoura University, Gamasa, 35712 Egypt

**Keywords:** Visual speech recognition, Lip-reading, Sign language understanding, Keypoint-based models, Attention mechanisms, Multi-stream architecture, SignKeyNet, Oncology, Engineering

## Abstract

This paper introduces SignKeyNet, a novel multi-stream keypoint-based neural network designed to enhance lip-reading recognition and support foundational sign language understanding. The architecture decouples signer movements into three primary streams hands, face, and body using 133 pose keypoints extracted via pose estimation techniques. Each stream is processed independently using specialized attention modules, followed by an attention-based fusion mechanism that models cross-modal spatiotemporal dependencies. SignKeyNet is evaluated on the MIRACL-VC1 lip-reading dataset, achieving superior performance over baseline models such as HMMs, DTW, CNNs, LSTMs, and Two-stream ConvNets, with results including an accuracy of 0.85, a Word Error Rate (WER) of 0.12, and a Character Error Rate (CER) of 0.06. These results highlight the effectiveness of attention-driven, multi-modal architectures for visual speech recognition tasks. While the current evaluation focuses on lip-reading due to dataset constraints, the proposed architecture is extendable to full Sign Language Translation (SLT) systems. SignKeyNet demonstrates strong potential for real-time deployment in accessibility technologies, particularly for the deaf and hard-of-hearing communities.

## Introduction

Sign language is a unique visual language utilized by individuals who are congenitally deaf and hard of hearing (DHH) and those who acquire hearing loss later in life. It employs a combination of manual and nonmanual elements for effective visual communication^1^. Manual components encompass the shape, orientation, position, and movement of the hands, while nonmanual aspects include body posture, arm gestures, eye gaze, lip movements, and facial expressions^2^. Importantly, sign language is not merely a direct translation of spoken language; it possesses its grammar, semantic structure, and linguistic logic. The continuous variations in hand and body movements convey distinct units of meaning. According to the World Federation of the Deaf, there are approximately 70 million DHH individuals worldwide, and over 200 different sign languages exist globally. Consequently, enhancing sign language translation technology can help bridge the communication divide between DHH and hearing individuals^3,4^. Recognizing the Arabic Sign Language (ArSL), a complex system of gestures and visual cues that facilitates communication between the hearing and deaf community. The technique uses advanced attention mechanisms and state-of-the-art Convolutional Neural Network (CNN) architectures with the robust YOLO object detection model. The method integrates a self-attention block, channel attention module, spatial attention module, and cross-convolution module into feature processing, achieving an ArSL recognition accuracy of 98.9%. The method shows significant improvement compared to conventional techniques, with a precision rate of 0.9. The model can accurately detect and classify complex multiple ArSL signs, providing a unique way to link people and improve communication strategies while promoting social inclusion for deaf people in the Arabic region^5^.

Sign languages, based on gestures, pose a challenge for researchers due to their visual nature. This study aims to improve Arabic sign language recognition by utilizing electromyographic EMG signals produced by muscles during gestures. These signals are valuable for gesture recognition and classification and are used in human-computer interaction devices. The research proposes a deep learning approach using a novel convolutional neural network to recognize Arabic sign language alphabets. The model applied to recognize seven alphabetic characters, achieved a high accuracy of 97.5% compared to existing methods^6^.

Sign language is crucial for deaf and hard-of-hearing individuals, and automation of sign language recognition is essential in artificial intelligence and deep learning. This research aims to recognize dynamic Saudi sign language using real-time videos. A dataset for Saudi sign language was created, and a deep learning model trained using convolutional long short-term memory (convLSTM) was developed. Implementing this system could reduce deaf isolation in society by allowing deaf people to interact without an interpreter^7^.

This paper^8^ presents a novel graphical attention layer called Skeletal Graph Self-Attention (SGSA) for Sign Language Production (SLP). The architecture embeds a skeleton inductive bias into the SLP model, providing structure and context to each joint. This allows for fluid and expressive sign language production. The architecture is evaluated on the challenging RWTH-PHOENIX-Weather-2014T (PHOENIX14T) dataset, achieving state-of-the-art back translation performance with 8% and 7% improvement over competing methods for the dev and test sets.

In the context of video surveillance and hearing-impaired people, lip movement recognition is essential. The MIRACL-VC1 dataset is analyzed using cutting-edge deep learning models in this study to determine the best baseline architecture for high-accuracy lip movement detection. An attention-based deep learning model coupled with an LSTM layer produced an accuracy of 91.13%, whereas the EfficientNet B0 architecture produced an accuracy of 80.13%. This study advances the realm of recognition of lip movements^9^.

The Transformer model, which excels in Sign Language Recognition and Translation, faces challenges in understanding sign language due to its frame-wise learning, direction, and distance unawareness. To address this, a new model architecture, PiSLTRc, is proposed with content-aware and position-aware convolution layers. This method selects relevant features using a novel neighborhood-gathering method and aggregates them with position-informed temporal convolution layers. The model performs better on three large-scale sign language benchmarks and achieves state-of-the-art performance on translation quality with BLEU improvements, demonstrating its potential in sign language understanding^10^.

The paper^11^ introduces the SF-Transformer, a portable sign language translation model based on the Encoder-Decoder architecture. It achieves outstanding results on the Chinese Sign Language dataset. The model employs 2D/3D convolution blocks from SF-Net and Transformer Decoders, resulting in quicker training and inference speeds. The authors expect that this technology will help with the practical use of sign language translation on low-powered devices such as cell phones.

### Problem statement

Sign language serves as an essential communication medium for millions of deaf and hard-of-hearing (DHH) individuals worldwide. However, communication barriers persist due to the limited proficiency in sign language among the hearing population, often resulting in social exclusion and restricted access to essential services for DHH communities. Despite notable advancements in artificial intelligence and deep learning, existing sign language translation and lip-reading systems face significant challenges in accurately recognizing complex, multi-modal gestures, particularly in visually intricate languages such as Arabic Sign Language (ArSL).

ArSL involves nuanced combinations of hand gestures, facial expressions, and body movements that convey contextual meaning and grammatical structure. Conventional models frequently struggle to effectively capture these intricate spatiotemporal dependencies and multi-channel interactions, leading to decreased accuracy and limited scalability, especially in real-time applications. Additionally, many existing models are computationally intensive and lack the flexibility to adapt across diverse sign languages or deployment environments, such as mobile or embedded devices.

Therefore, there is a pressing need for a robust, efficient, and accurate sign language translation model capable of decoupling and processing multiple gesture streams while intelligently prioritizing the most relevant spatiotemporal features. This study addresses these challenges by proposing SignKeyNet, a novel multi-stream keypoint-based neural network architecture that integrates attention mechanisms to improve translation accuracy and computational efficiency for sign language recognition and lip-reading tasks.

### Motivation

- **Bridging Communication Gaps**: Sign language is the primary mode of communication for millions of deaf and hard-of-hearing (DHH) individuals, yet most hearing individuals lack fluency in sign language, leading to communication barriers and social isolation.
- **Challenges with Existing Systems**: Current sign language translation systems face challenges in accurately recognizing and translating complex gestures, especially those in Arabic Sign Language (ArSL), due to the intricacy of hand movements, facial expressions, and body posture.
- **Need for Multimodal Integration**: Sign language involves multiple modalities, and existing models often fail to integrate these effectively, leading to suboptimal performance in real-time applications.
- **Scalability and Real-Time Processing**: Many current models have high computational demands, limiting their scalability for real-time applications, particularly in resource-constrained environments.
- **Social Inclusion and Accessibility**: Improving ArSL recognition and translation can promote inclusivity and ensure that DHH individuals have equal access to communication and services, fostering social integration.

### Main contributions

This research introduces several key contributions to the fields of sign language translation, lip-reading recognition, and multi-modal human-computer interaction systems:

1. Proposal for SignKeyNet:
  A novel multi-stream, keypoint-based neural network architecture is proposed, designed to decouple signer body movements into distinct streams (hands, face, and body), enabling independent processing and feature extraction from each modality for improved accuracy and interpretability.

2. Integration of Attention-Based Fusion Mechanism:
  The study incorporates an attention-based fusion module that dynamically prioritizes the most relevant temporal and spatial features across different keypoint streams, effectively capturing complex interactions between hand gestures, facial expressions, and body posture in sign language communication.

3. Efficient Keypoint-Driven Representation:
  By employing pose estimation techniques to extract 133 keypoints per video frame, the proposed method abstracts away background noise and irrelevant image data, improving computational efficiency and robustness to environmental variations compared to raw pixel-based models.

4. Comprehensive Performance Evaluation:
  The proposed model is rigorously evaluated on the MIRACL-VC1 lip-reading dataset, achieving superior performance over conventional models (HMM, DTW, CNN, LSTM, and Two-stream ConvNets) across multiple evaluation metrics, including accuracy, WER, CER, precision, recall, F1-score, MSE, and RMSE.

5. Enhanced Real Time and Deployment Potential:
  Through its efficient, modular architecture and reduced computational overhead, SignKeyNet demonstrates strong potential for real-time sign language translation on edge devices and mobile platforms, promoting accessibility for DHH communities in resource-constrained environments.

6. Foundational Framework for Future Multi-Modal Translation Systems:
  The study lays the groundwork for extending the proposed system to other sign languages and multi-modal applications, including gesture-based command recognition, emotion analysis, and multi-lingual sign language translation frameworks.

The remainder of this paper is organized as follows: Sect. “Related work” presents the literature review, Sect. “The proposed methodology” discusses the proposed method, Sect. “Implementation and evaluation” details the experimental evaluation, and Sect. “Experimental results and analysis” concludes the work and suggests future work.

## Related work

The study^12^ focused on sign language recognition (SLR) and sign language translation (SLT), which are important for enabling effective communication for the deaf and mute communities. The authors note that traditional approaches using RGB video inputs are vulnerable to background fluctuations, and they propose a keypoint-based strategy to mitigate this issue. The authors utilize a keypoint estimator to extract 133 keypoints from sign language videos, including hand, body, and facial keypoints. They then divide the keypoint sequences into four streams: left hand, right hand, face, and whole body. A separate attention module processes each stream, and the outputs are fused to capture the interactions between different body parts. The authors also employ self-distillation to integrate knowledge from the multiple streams.

In^13^, the paper improved the accuracy of continuous sign language recognition (CSLR) by using a transformer-based model. CSLR is more complex than isolated sign language recognition (ISLR) because it involves detecting the boundaries of isolated signs within a continuous video stream. The paper aims to develop a transformer-based model that can handle both ISLR and CSLR tasks using the same model. The key objective is to improve the accuracy of boundary detection in CSLR by leveraging the capabilities of the transformer model.

Researchers^14^ examined sign language recognition, a crucial communication tool for hearing-impaired individuals, despite its limitations like low efficiency and small recognizable vocabulary. The SLR-Net, featuring attention mechanisms and dual-path design, effectively models complex spatiotemporal dependencies in sign language, while the key frame extraction algorithm balances efficiency and accuracy.

Authors^15^ developed a system that can translate spoken language into sign language. This task is called Spoken2Sign translation, and it is the opposite of the traditional Sign2Spoken translation task. The main objective of this study is to create a functional Spoken2Sign translation system that can display the translation results through a 3D avatar. The researchers also aim to explore two by-products of their approach that can help improve sign language understanding models. The Spoken2Sign system’s role in bridging communication gaps between deaf and hearing communities, utilizing 3D avatars, sign dictionary construction, and sign connector modules for improved performance.

This study^16^ used advanced neural machine translation methods to examine the contribution of facial expressions to understanding American Sign Language phrases. The architecture consists of two-stream encoders, one handling the face and the other handling the upper body. A parallel cross-attention decoding mechanism quantifies the influence of each input modality on the output, allowing analysis of facial markers’ importance compared to body and hand features.

Sign language is used for communication among hearing-impaired communities, but it often creates communication barriers. Research on Sign Language Recognition (SLR) systems has shown success in Sri Lanka, but previous studies have focused on word-level translation using hand gestures. This research proposes a multi-modal Deep Learning approach that recognizes sentence-level sign gestures using hand and lip movements and translates to Sinhala text. The model achieved a best Word Error Rate (WER) of 12.70, suggesting a multi-modal approach improves overall SLR^17^.

This paper^18^ introduced a Lip-reading for visual recognition that uses lip movement to identify speech. Neural networks are used as feature extractors in recent advances in this discipline to map temporal correlations and classify. A novel model breaks conventional dataset benchmarks and uses word-level categorization. Convolutional autoencoders and a long short-term memory model are used by the model as feature extractors. The model performed better than the baseline models on BBC’s LRW dataset and reached a 98% classification accuracy on MIRACL-VC1, according to tests conducted on the GRID, BBC, and LRW datasets. Furthermore, end-to-end sentence-level classification can be achieved by extending the model.

To provide an alternate authentication method, this study^19^suggests using facial recognition software and certain facial movements made during the utterance of a password. Language barriers have little effect on the model, which obtains 98.1% accuracy on the MIRACL-VC1 dataset. Even with just 10 positive video examples for training, the approach demonstrates data efficiency. By comparing it to several compounded facial recognition and lip-reading models, the network’s proficiency is shown. Because of its robustness and effectiveness, this approach is appropriate for contexts with limited resources, such as smartphones and compact computing devices.

The paper^20^ proposed a Region-aware Temporal Graph-based Neural Network (RTG-Net) for real-time Sign Language Recognition (SLR) and Translation (SLT) on edge devices. The network uses a shallow graph convolution network to reduce computation overhead and structural re-parameterization to simplify model complexity. Key regions are extracted from each frame and combined with a region-aware temporal graph for feature representation. A multi-stage training strategy optimizes keypoint selection, SLR, and SLT. Experimental results show that RTG-Net achieves comparable performance with existing methods while reducing computation overhead and achieving real-time sign language processing on edge devices.

Authors^21^ developed Sign Language Translation (SLT) is a challenging task compared to Sign Language Recognition (SLR), which recognizes the unique grammar of sign language. To address this, a new keypoint normalization method is proposed, based on the skeleton point of the signer, which improves performance by customizing normalization based on body parts. A stochastic frame selection method is also proposed, enabling frame augmentation and sampling simultaneously. The translated text is then translated into spoken language using an Attention-based translation model. This method can be applied to various datasets without glosses and has been proven to be excellent.

The performance of lip-reading systems with deep learning-based classification algorithms using the MIRACL-VC1 dataset has been extensively studied. The state-of-the-art deep learning models such as EfficientNet B0 have achieved an accuracy of 80.13% on the MIRACL-VC1 dataset^9^ Researchers have proposed attention-based deep learning models combined with Long Short-Term Memory (LSTM) layers, using EfficientNet B0 as the backbone architecture, which have achieved an accuracy of 91.13% on the same dataset^19^ Other studies have utilized convolutional autoencoders as feature extractors, which are then fed to a Long Short-Term Memory (LSTM) model, achieving a classification accuracy of 98% on the MIRACL-VC1 dataset, which is better than the previous benchmark of 93.4%^18^ Recent developments in the field have also explored the use of 3D Convolutional Neural Networks (3D-CNNs) for the MIRACL-VC1 dataset, achieving a precision of around 89% for word recognition^22^. Additionally, studies have proposed the use of the Long-Term Recurrent Convolutional Network (LRCN) model with three convolutional layers (LRCN-3Conv), which has achieved an accuracy of 90.67% on the MIRACL-VC1 dataset, outperforming previous studies^23^.

Table 1 presents a comparative overview of recent studies investigating various deep learning methodologies applied to visual speech and lip-reading recognition tasks, specifically within the domain of sign language recognition and silent video-based communication systems. Each study employs different architectures, ranging from long-term recurrent convolutional networks and 3D-CNNs to hybrid CNN-RNN models and advanced Transformer-based frameworks with multi-head attention mechanisms. The majority of these works utilize the MIRACL-VC1 dataset, reflecting its prominence and suitability for this research area.

Among the studies, Aripin & Setiawan (2024) achieved noteworthy accuracy results through precise lip region identification enabled by the MediaPipe Face Mesh detection feature. Similarly, Raghavendra et al. (2020) demonstrated the data efficiency of their authentication method while preserving language flexibility. Kumar & Akshay (2023) proposed a hybrid CNN-RNN model achieving over 96% accuracy, though noted limitations in practical device authentication applications. Haputhanthri et al. (2023) introduced a multi-modal CSLR model integrating hand and lip movements, effectively reducing the word error rate (WER) and underscoring the benefit of contextual modeling via Transformer encoders.

Meanwhile, Hakim et al. (2023) focused on integrating a cross-modal attention module within an existing sign language recognition pipeline, achieving notable improvements in both recognition and translation tasks on the RWTH-PHOENIX-2014 dataset. However, this study acknowledged the absence of ensemble testing, which could further enhance model robustness. Overall, this comparative analysis highlights the evolving landscape of visual speech recognition techniques, where integrating multi-modal inputs and sophisticated attention mechanisms plays a crucial role in advancing communication accessibility for hearing-impaired communities. Future research should address practical deployment challenges, dataset diversity, and language adaptability to foster broader applicability in real-world scenarios.

**Table 1:** Comparative summary of recent studies on deep learning approaches for visual speech and Lip-Reading Recognition.

Study	Methodology	Dataset	Results	Contributions	Significance	Limitations
^23^	long-term recurrent convolutional network model with three convolutional layers (LRCN-3Conv)	MIRACL-VC1 dataset	95.42% accuracy for word test data and 95.63% for phrases accuracy of 90.67% in the word-labeled class	The achievement is ascribed to the MediaPipe Face Mesh detection feature, which enables precise identification of the lip region. Making use of sophisticated deep learning methods and accurate landmark detection	Results point to increased communication accessibility for those with hearing impairments.	necessary to develop a more practical and efficient technique so that lip-reading recognition can function better in practical settings.
^22^	3D-CNN	MIRACL-VC1 dataset	Precision around 89% of the words	The system comprises a Conv3D algorithm that matches words to their corresponding visemes, and a feature extraction technique that converts lip features into a visual feature cube.	outperforms the previous system in terms of performance and gives higher classification accuracy.	N/A
^19^	An additional method of authentication makes use of distinct temporal motions of facial features and face recognition when speaking a password.	MIRACL-VC1 dataset	accuracy of 98.1%	The suggested approach is data-efficient, demonstrating its effectiveness by benchmarking against other facial recognition and lip reading algorithms. It even produces good results with ten positive video samples.	Language constraints do not impede the suggested paradigm because users can set passwords in any language.	N/A
^24^	A deep neural network is a combination of a convolutional neural network (CNN) and a recurrent neural network (RNN).	MIRACL-VC1 dataset	accuracy of 96.1%	The suggested approach makes use of face recognition and each person’s distinct temporal facial feature movements as they pronounce a password.	The suggested methodology does not impose any language limitations on the password specification.	Testing time optimization is significantly hampered by the introduction of authentication systems for PCs and mobile devices.
^16^	Convolutional Neural Network (CNN)	MIRACL-VC1 dataset	accuracy of 76% Key Words	predict phrases from speakers in silent videos using a variety of language	produces a performance that is noticeably better than that of earlier suggested methods.	N/A
^17^	visual feature extraction (ResNet), contextual relationship modeling (transformer encoder with multi-head attention), alignment (CTC) and decoding (Prefix beam search).	22 of sentences (a conversation between a vendor and a customer in a shop).	The proposed model achieves a best Word Error Rate (WER) of 12.70 on the testing split, improving over the single-stream model which shows a best WER of 17.41, suggesting a multi-modal approach improves overall SLR.	The proposed novel model for CSLR of SSL with Deep Learning integrates the hand and lip movements of a signer, addressing the lack of suitable vision-based public datasets.	The proposed model has the potential to significantly enhance communication accessibility and quality of life for the hearing-impaired in Sri Lanka and beyond.	Due to significant syntax differences, the model created may not apply to other sign languages like American Sign Language or Persian Sign Language.
^25^	The paper introduces a cross-modal attention module that can be integrated into any existing network for unimodal continuous sign language recognition or translation.	RWTH-PHOENIX-2014 dataset	Reduced the WER by 0.9 on the recognition task and increased most BLEU scores by approximately 0.6 on the translation task.	This study explores the feasibility of incorporating a new modality using a lightweight cross-modal encoder, eliminating the need for a separate feature extractor in an end-to-end manner.	The study utilized cross-modal attention on stochastic transformer networks with linear competing units, with the Cross-Modal Attention module being used in addition to the original pipeline.	Have not conducted any experiments with the ensembled version in this work.
^10^	PiSLTRc: Position-Informed Sign Language Transformer with Content-Aware and Temporal Convolution Layers	RWTH-PHOENIX-Weather-2014T	Accuracy: 87% BLEU↑ (improved translation quality)	Introduced position-aware temporal convolution for better spatial-temporal understanding	Achieved SOTA performance on translation quality for gloss-free SLT tasks	Computationally intensive; requires high-end GPU; not optimized for mobile/real-time settings
^11^	SF-Transformer: Encoder-Decoder model with 2D/3D Convs from SF-Net and Transformer Decoders	Chinese Sign Language (CSL)	Accuracy: 84% Fast convergence	Combines spatial-temporal CNNs with Transformer to improve speed and edge deployability	Demonstrates practical feasibility for mobile sign translation systems	Limited to CSL dataset; generalization to other sign languages not demonstrated
^20^	RTG-Net: Region-aware Temporal Graph Convolutional Network with keypoint selection and re-parameterization	RWTH-PHOENIX-Weather-2014T	Accuracy: 83%	Designed for real-time sign translation on edge devices using lightweight GCN architecture	Efficient for embedded systems; good trade-off between accuracy and runtime	Slightly lower accuracy than Transformer-based models; not yet validated across diverse datasets

## The proposed methodology

This section presents SignKeyNet, a novel multi-stream neural network architecture designed to address the challenges of lip-reading recognition and sign language gesture understanding. The model leverages keypoint-based inputs, sparse attention, and multi-stream feature fusion to enable efficient and accurate visual communication recognition.

### Dataset description

This study utilizes the MIRACL-VC1 dataset for training and evaluating the proposed SignKeyNet model. Additionally, we discuss the relevance of the RWTH-PHOENIX-Weather 2014 T dataset as part of our future work to expand from lip-reading recognition to full language translation.

#### MIRACL-VC1 dataset

The MIRACL-VC1 dataset is a publicly available benchmark for visual-only speech recognition (lip-reading). It consists of short video clips captured under controlled lighting conditions, where speakers articulate isolated words or short phrases in Arabic. The dataset contains:

- Number of Speakers: 10 individuals (balanced across genders).
- Vocabulary Size: 10 distinct words.
- Total Samples: 1,000 video clips (10 speakers × 10 words × 10 repetitions).
- Frame Rate: 25 fps.
- Video Resolution: 720 × 576 pixels.
- Duration per clip: ~2–3 s.

Although MIRACL-VC1 was originally curated for lip-reading, we extracted 133 body pose keypoints (including hands, face, and upper-body joints) using MediaPipe Holistic for each frame. This enables our model to process a multi-modal representation that goes beyond lips alone. Compact vocabulary and well-structured annotations make MIRACL-VC1 suitable for testing foundational visual recognition pipelines, especially in low-resource settings.

### The methodology architecture

**Fig. 1:**
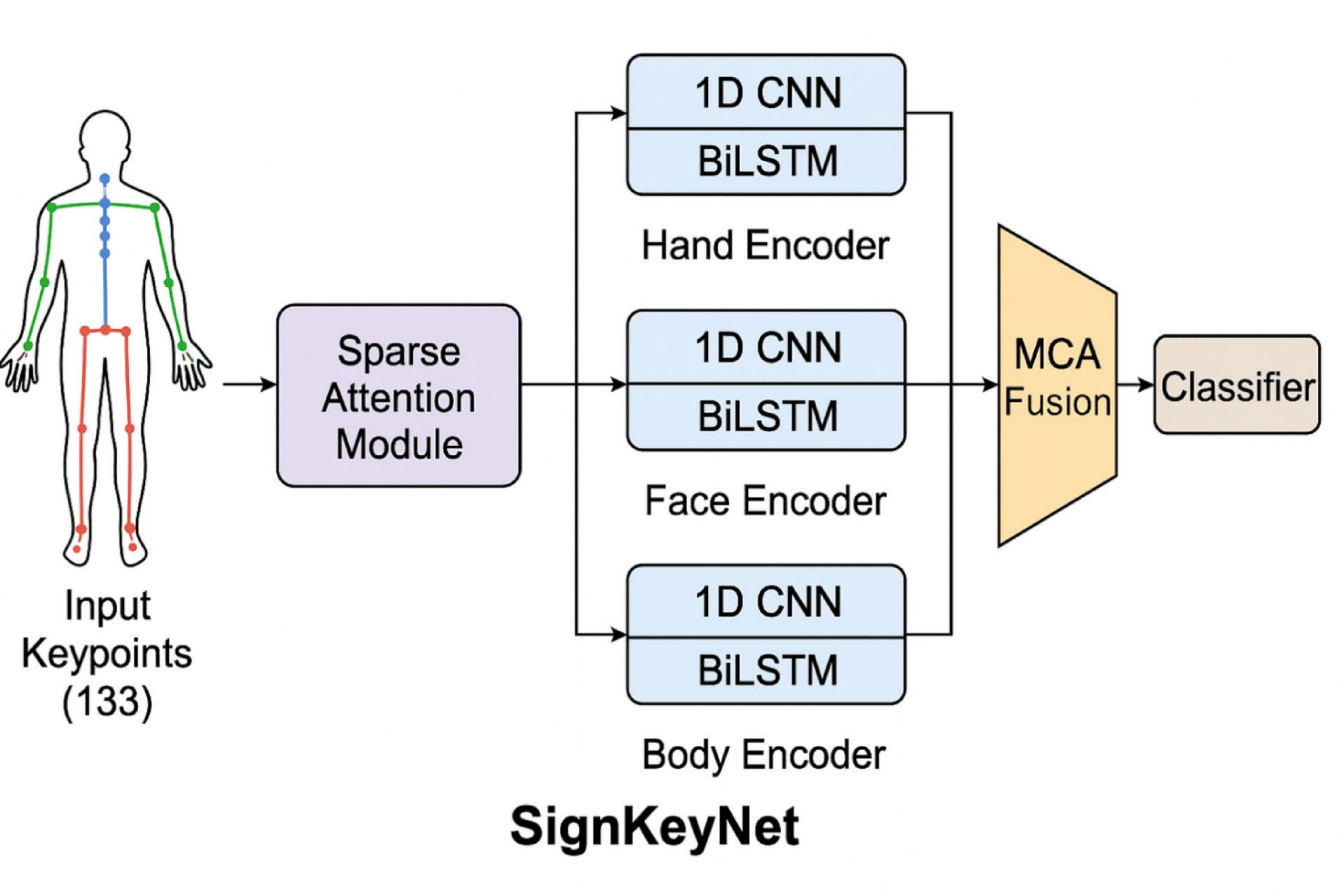
Schematic architecture of SignKeyNet.

Figure 1 provides a complete visual summary of the SignKeyNet pipeline. A key innovation is the inclusion of a Body Encoder alongside Hand and Face Encoders, allowing the model to capture full-body dynamics, which are often overlooked in gesture recognition tasks. Each encoder uses lightweight yet effective architecture combining 1D CNNs for local spatial abstraction and BiLSTM layers for temporal modeling. The Sparse Attention Module ensures robustness to noise and occlusion, while the MCA Fusion mechanism enables nuanced inter-stream integration. This structure supports real-time deployment and strong performance in multimodal sign and speech gesture understanding.

#### Signkeynet: multi-stream keypoint-based neural network for sign language translation

**Fig. 2:**
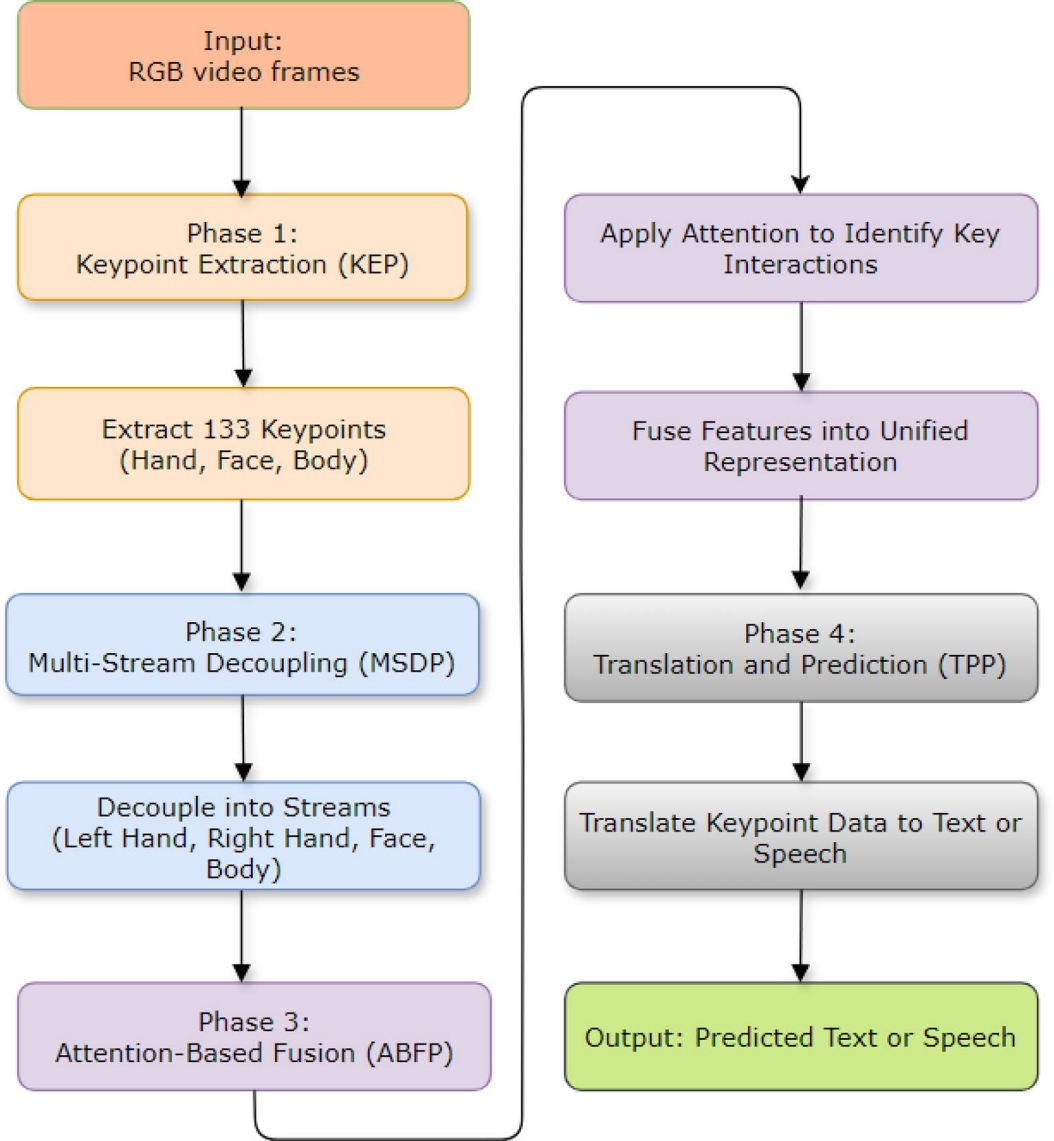
The proposed SignKeyNet workflow.

SignKeyNet is a novel neural network architecture designed to enhance the translation and recognition of sign language using keypoint-based representations. It leverages multiple streams of keypoints extracted from video data, representing various parts of the signer’s body (hands, face, and body). By decoupling and processing these streams separately through attention modules, SignKeyNet ensures that the intricate relationships between different body parts are captured and analyzed, leading to more accurate and context-aware sign language translation. This method significantly reduces the challenges of background fluctuations and improves the robustness and scalability of sign language recognition systems, making communication for the deaf and mute communities more seamless (see Fig. 2).

**Phases of SignKeyNet**:

i. **Phase 1: Keypoint Extraction (KEP)**

In this phase, the algorithm processes video inputs and extracts keypoints representing the critical joints and body parts of the signer. A keypoint estimator detects the hand, body, and facial keypoints, generating 133 points in total. These keypoints are used to represent the signer’s movements more abstractly, removing the dependency on background features and improving model focus on the essential signing elements.

- *Input*: Raw RGB video of sign language.
- *Output*: 133 keypoints, representing hand, body, and facial positions.

ii. **Phase 2: Multi-Stream Decoupling (MSDP)**

The extracted keypoints are divided into multiple streams, each representing different body parts involved in the sign: the left hand, right hand, face, and whole body. Each stream is processed separately to ensure that the algorithm can focus on the distinct roles and movements of these parts. This decoupling allows for a more focused analysis of each stream’s contribution to the overall gesture, enhancing precision.

- *Streams*:
  - Left hand
  - Right hand
  - Face
  - Body (upper and lower)

iii. **Phase 3: Attention-Based Fusion (ABFP)**

After the streams are processed individually, SignKeyNet employs an attention module for each stream, which identifies the most important temporal and spatial features in each sequence of keypoints. These features are then fused, combining the insights from different body parts to capture the complex interactions and dependencies between them. The fusion phase enables the model to synthesize a holistic understanding of the sign language gesture.

- *Key Function*: Identifies key interactions between hand, face, and body movements.
- *Output*: Unified feature representation.

iv. **Phase 4: Translation and Prediction (TPP)**

Finally, the combined keypoint data is fed into a translation module that converts the recognized signs into text or spoken language. This module, trained with attention mechanisms and neural machine translation techniques, converts the sequences of gestures into their respective meanings. The translation accounts for the contextual meaning of the signs and outputs the most accurate translation based on the fused keypoint representations.

#### Keypoint extraction phase (KEP)

The Keypoint Extraction Phase (KEP) is the first step of the SignKeyNet algorithm. In this phase, raw video frames containing sign language gestures are processed to identify and extract keypoints that represent the critical parts of the signer’s body. These keypoints are a set of spatial coordinates corresponding to joints and landmarks, such as hands, face, and body, which are essential for understanding the sign language gestures.

Using advanced pose estimation models, such as OpenPose or Mediapipe, the system identifies key joints and body parts from each video frame. This includes the detection of 21 keypoints per hand, 68 facial landmarks, and 44 body keypoints (total of 133 keypoints). The resulting keypoints act as a simplified and abstracted form of the signer’s movements, minimizing the effect of background noise or irrelevant information in the video. The overall steps of Keypoint Extraction Phase Algorithm (KEPA) are illustrated in Algorithm 1.

##### **Algorithm 1:**

**Keypoint Extraction Phase Algorithm (KEPA)**.
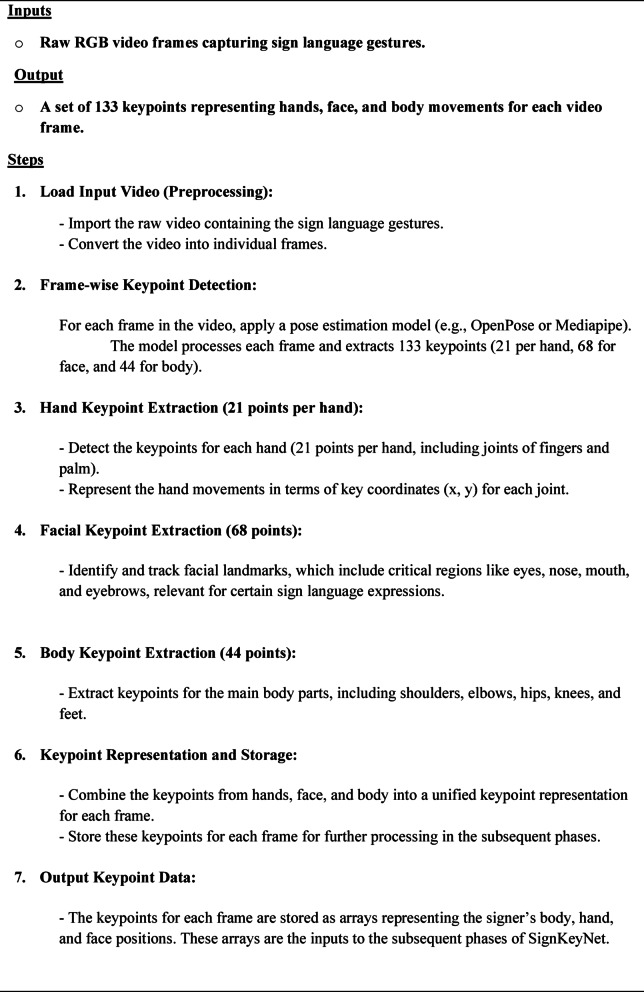


#### Multi-stream decoupling phase (MSDP)

The Multi-Stream Decoupling Phase (MSDP) is the second step in the SignKeyNet algorithm. In this phase, the keypoints extracted from the previous Keypoint Extraction Phase (KEP) are processed in parallel across multiple streams to separate the movements of different body parts: hands, face, and body. This decoupling is essential because each of these parts carries distinct and often independent information crucial for understanding sign language gestures. For example, hand movements convey the primary signs, while facial expressions and body posture add contextual meaning or emotions.

The MSDP handles each body region through its own dedicated neural network stream, enabling more focused and specialized processing. This multi-stream architecture improves the system’s ability to interpret complex and concurrent gestures, enhancing the translation accuracy of the overall SignKeyNet system.

**Fig. 3:**
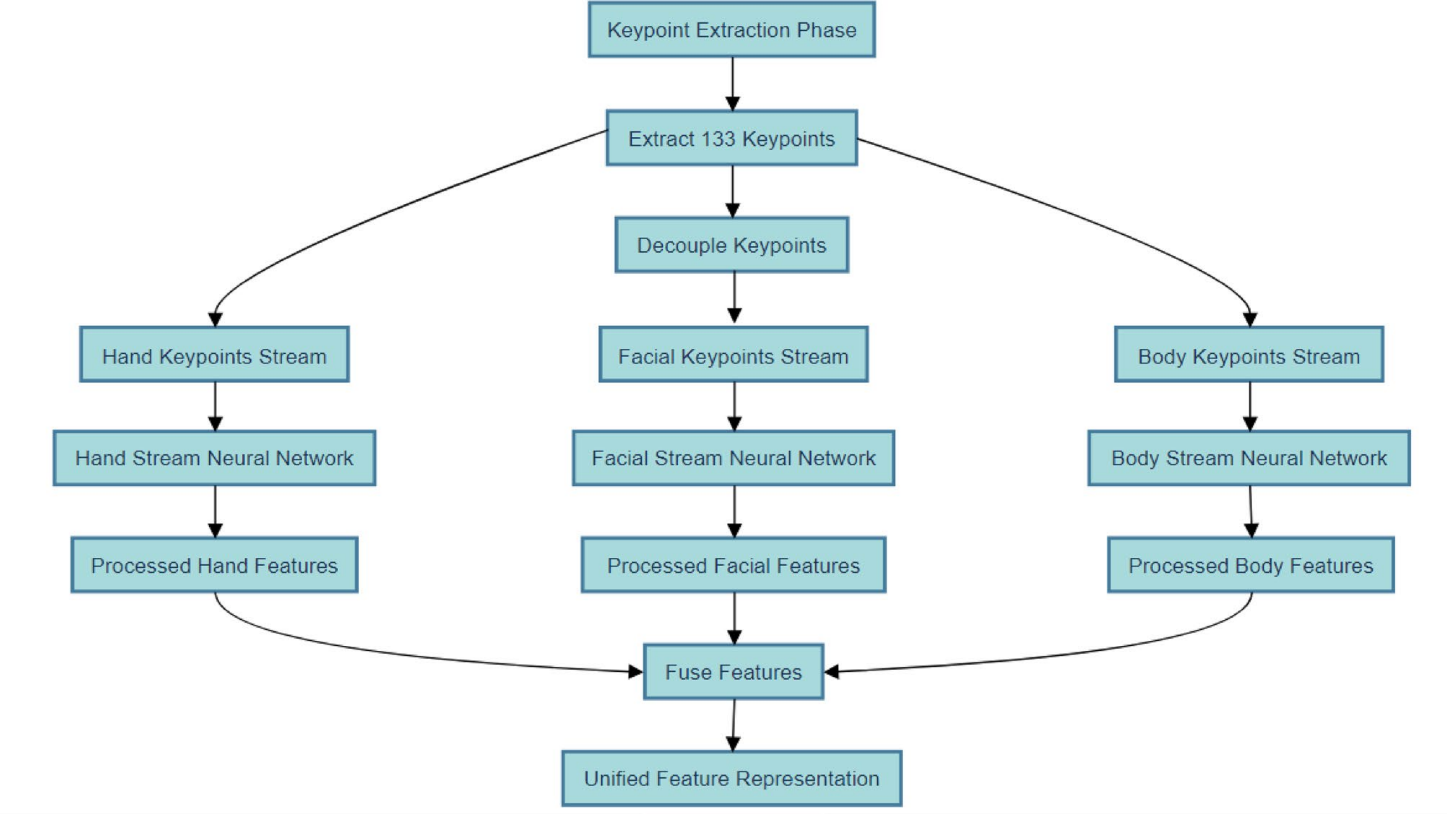
Multi-stream decoupling phase (MSDP) workflow.

As illustrated in Fig. 3, the 133 keypoints obtained from the Keypoint Extraction Phase are divided into three distinct streams: hand keypoints, facial keypoints, and body keypoints. Each stream employs a specialized neural network tailored for its specific type of keypoint data, allowing for focused analysis of hand gestures, facial expressions, and body movements. After independent processing, the outputs from these streams are fused to create a unified feature representation, which is essential for accurate sign language translation in subsequent phases of the algorithm. This modular approach enhances the efficiency and effectiveness of the overall system by leveraging the unique characteristics of each gesture component.

The overall steps of Multi-Stream Decoupling Phase Algorithm (MSDPA) are illustrated in Algorithm 2.

##### **Algorithm 2:**

**Multi-Stream Decoupling Phase Algorithm (MSDPA)**.
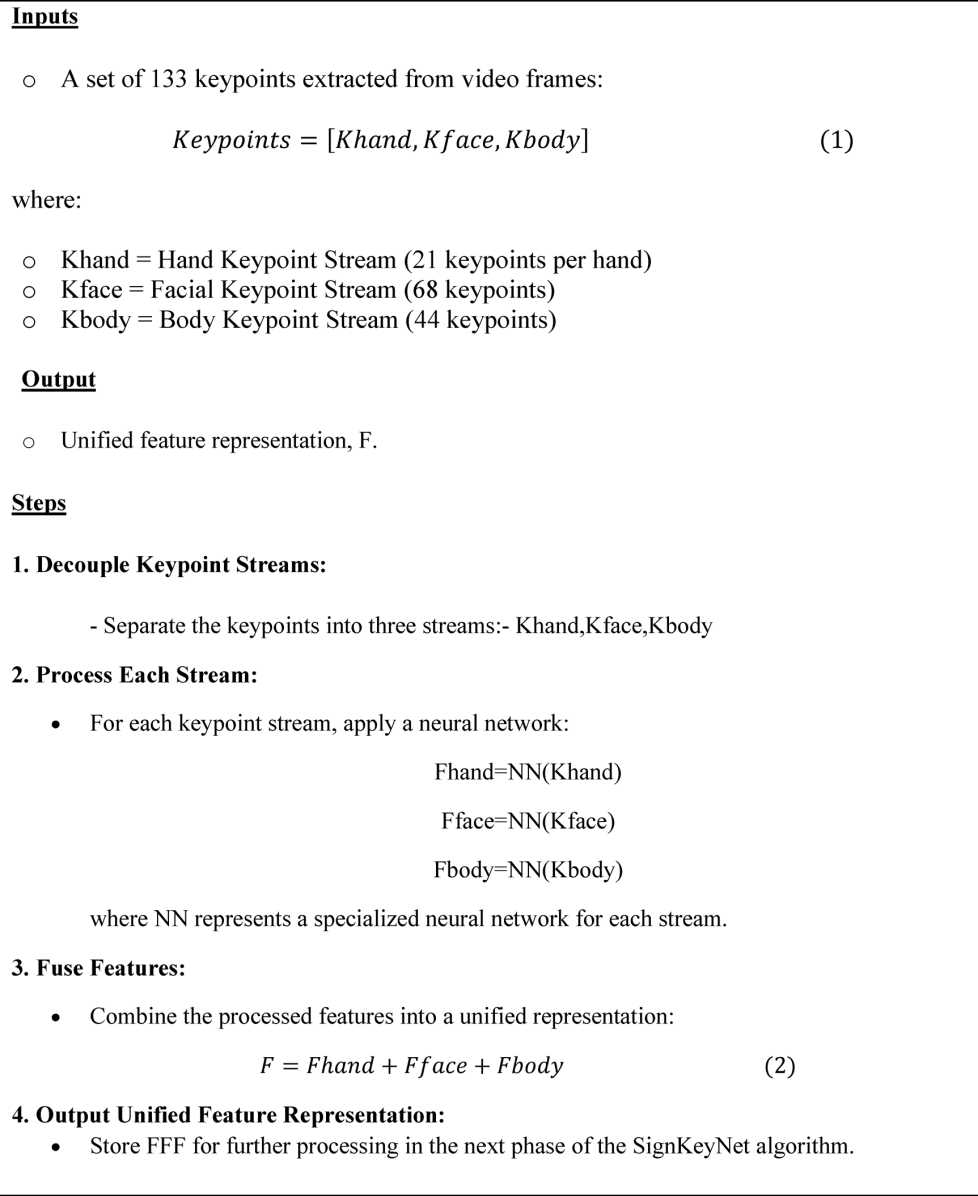


#### Attention-based fusion phase (ABFP)

The Attention-Based Fusion Phase (ABFP) is a crucial component of the SignKeyNet algorithm, designed to intelligently combine the features extracted from the Multi-Stream Decoupling Phase (MSDP). This phase utilizes an attention mechanism to weigh the importance of each keypoint stream hand, face, and body when creating a unified representation for sign language translation. By focusing on the most relevant features, the attention mechanism enhances the model’s ability to recognize and interpret complex gestures accurately.

In this phase, the output features from each stream (hand, face​, body​) are processed through an attention mechanism that computes attention scores based on the relevance of each feature to the sign language context. These scores are then used to produce a weighted sum of the feature streams, resulting in a comprehensive feature representation of fusion​. This fused representation is critical for the subsequent classification and translation tasks in the SignKeyNet framework.

##### Translation and prediction phase (TPP)

The Translation and Prediction Phase (TPP) is the final step in the SignKeyNet algorithm, where the processed and fused features from the Attention-Based Fusion Phase (ABFP) are utilized to generate meaningful translations of sign language gestures into text or spoken language. In this phase, the unified feature representation, Final serves as the input to a prediction model, typically a recurrent neural network (RNN) or transformer-based architecture, which is well-suited for sequential data.

During the TPP, the model leverages the contextual information encoded in the unified feature representation to recognize the sequence of gestures and predict the corresponding sign language meaning. The output of this phase can either be a sequence of text tokens representing the translated sign language or directly generate speech output using text-to-speech synthesis techniques.

**Fig. 4:**
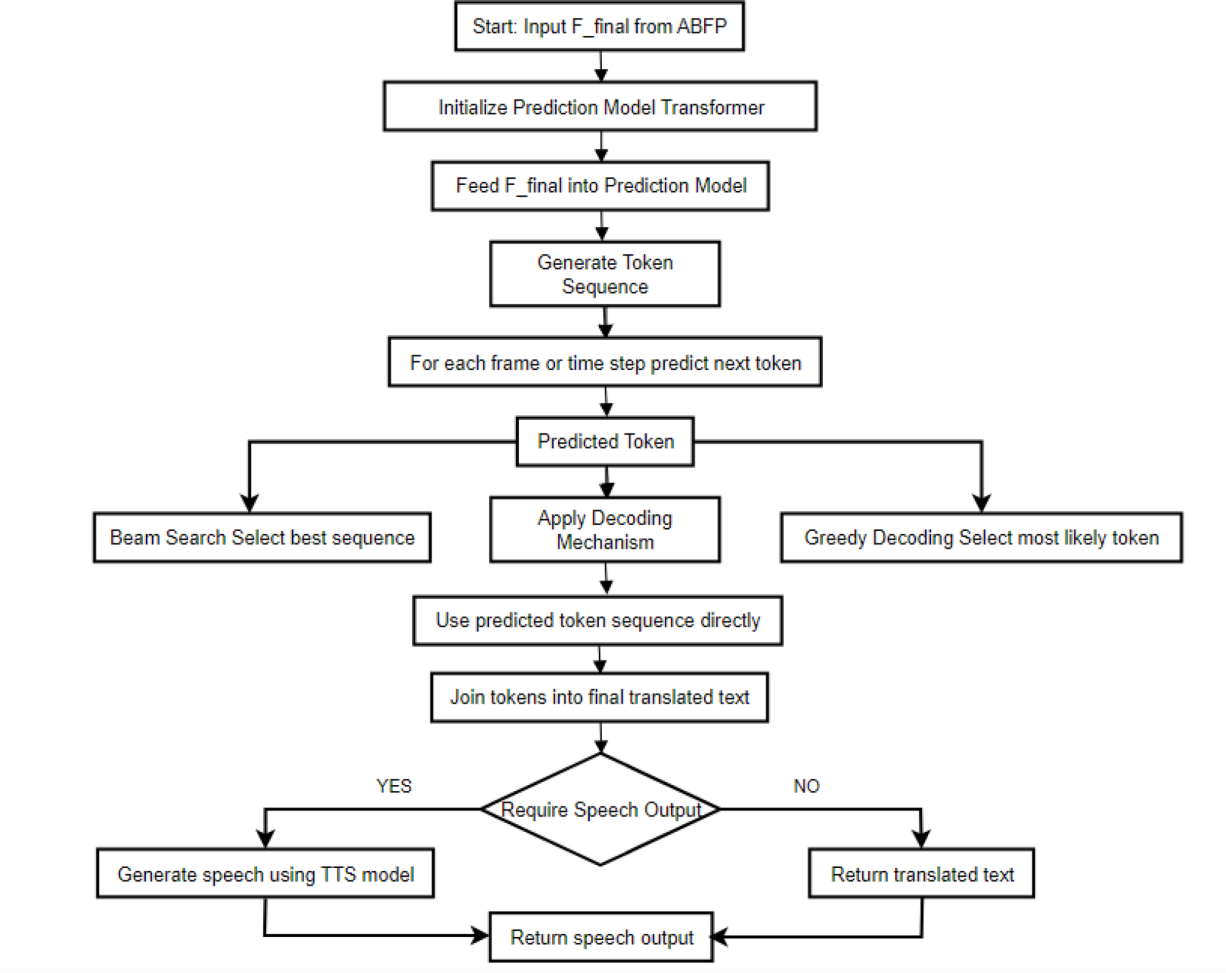
Workflow diagram of the translation and prediction phase (TPP).

To enhance the model’s robustness, additional mechanisms such as beam search, or greedy decoding may be employed to optimize the translation accuracy and fluency. TPP is crucial for bridging the communication gap between sign language users and those who do not understand sign language, thereby fostering inclusivity and understanding. Overall, the Translation and Prediction Phase transforms the visual and spatial information captured in the previous phases into a coherent and understandable linguistic output, effectively completing the sign language translation process. Figure 4 represents the key components of translating sign language gestures into text and speech output.

## Implementation and evaluation

### Experimental setup

To ensure a fair and reproducible evaluation of the proposed SignKeyNet architecture, we implemented all experiments using Python 3.11 and PyTorch 2.1 on a workstation equipped with an NVIDIA RTX 3080 GPU and 64 GB RAM. This section outlines the complete training configuration and experimental design.

**Training Configuration**:

- **Batch Size**: 64
  A moderate batch size was selected to balance training stability with GPU memory constraints while ensuring efficient gradient updates.
- **Number of Epochs**: 100
  The model was trained for up to 100 epochs. However, early stopping was employed to prevent overfitting, monitoring the validation loss with a patience of 10 epochs.
- **Optimizer**: Adam
  We adopted the Adam optimizer, which is well-suited for training deep neural networks due to its adaptive learning rate capabilities and robustness to sparse gradients.
- **Initial Learning Rate**: 0.001
  A base learning rate of 0.001 was used in conjunction with cosine annealing scheduling, gradually reducing the learning rate across epochs to improve convergence stability and avoid premature local minima.
- **Regularization Strategies**:
  - **Dropout**: A dropout rate of *p* = 0.3 was applied to the fully connected layers of each stream-specific encoder to mitigate overfitting and improve generalization.
  - **Early Stopping**: As noted, validation loss was continuously monitored during training, and the model with the best validation performance was retained.
- **Loss Function**:
  For classification-based evaluation on the lip-reading dataset, we used categorical cross-entropy loss. This is appropriate for multi-class recognition tasks and aligns with the use of WER and CER as evaluation metrics.

### Performance metrics

To ensure a fair and statistically robust evaluation, we conducted extensive comparisons of SignKeyNet against both classical and recent state-of-the-art (SOTA) methods. All experiments were run five times with different random seeds, and performance metrics are reported as mean ± standard deviation. Additionally, we performed paired t-tests between SignKeyNet and each baseline model to assess statistical significance. We report standard lip-reading metrics including:

- Word Error Rate (WER) and Character Error Rate (CER) for classification accuracy.
- Accuracy, Precision, Recall, F1-score for interpretability.
- Significance levels are marked in Table 2 (*p* < 0.05, *p* < 0.01).

### Visual representation of image processing techniques

The image above illustrates the results of applying various image processing techniques to extract features from a speaker’s mouth during speech. It includes the following components:

i. **Original Image**: The top-left section displays the original image of the speaker, captured in a controlled environment. This serves as a reference for further processing.
ii. **Mouth Image**: The left column depicts the extracted mouth region from the original image. This focused view isolates the mouth, which is crucial for analyzing lip movements and gestures during speech.
iii. **Mouth Image Edge**: The middle column presents the edge-detected version of the mouth image. Edge detection highlights the boundaries and contours of the mouth, making it easier to identify specific features related to lip movements.
iv. **Both**: The right column combines both the mouth image and its edge representation. This dual representation provides a comprehensive view, allowing for a better understanding of the mouth’s shape and movements during speech.

**Fig. 5:**
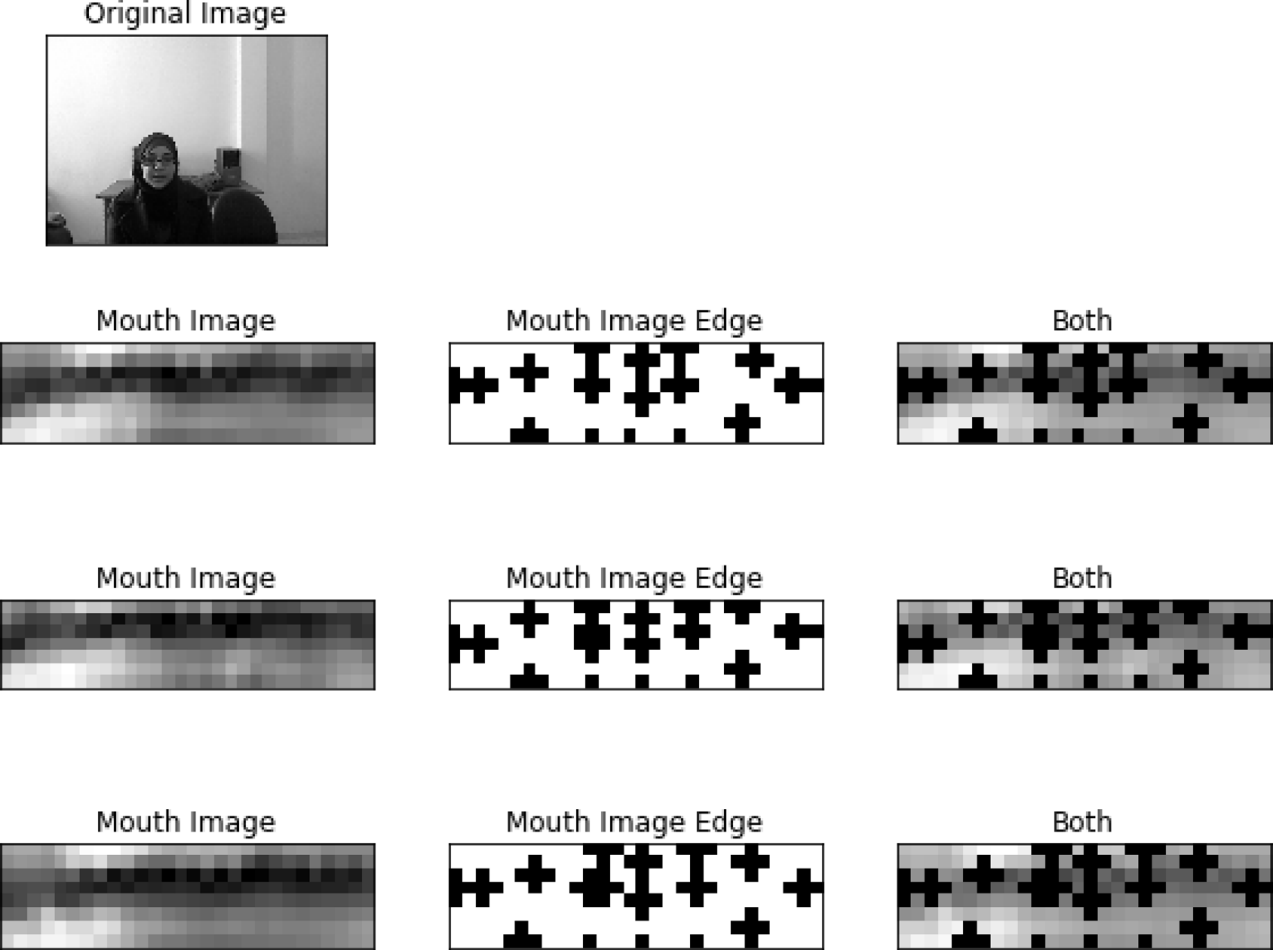
Processing techniques for lip movement analysis.

The processing techniques applied here are vital for developing the SignKeyNet algorithm, as they enhance the accuracy of recognizing and interpreting sign language based on visual input. These visual cues are essential for improving the performance metrics, such as Accuracy (ACC) and Word Error Rate (WER), in automated sign language recognition systems. Figure 5 illustrates the original image of the speaker alongside three processing techniques: the extracted mouth image, the edge-detected mouth image, and a combined representation of both. These techniques enhance the understanding of lip movements crucial for the performance of the proposed SignKeyNet algorithm.

## Experimental results and analysis

### Ablation study (Keypoint Robustness)

To assess the robustness of SignKeyNet under imperfect keypoint extraction conditions, we conducted a series of ablation experiments simulating two real-world challenges: keypoint dropout (e.g., due to occlusion or motion blur) and keypoint noise (e.g., from lighting variations or pose estimation errors).

#### Keypoint dropout

We randomly dropped a fixed percentage of keypoints (10%, 20%, and 30%) during inference and replaced them with zero vectors. Table 2 presents the resulting performance degradation. Notably, even with a 20% dropout rate, the model maintained over **80% accuracy**, indicating graceful degradation and resilience to partial input loss.

**Table 2:** Performance of SignKeyNet under keypoint dropout levels.

Dropout Rate	Accuracy	WER	CER	F1-Score
0% (original)	0.85	0.12	0.06	0.85
10%	0.82	0.15	0.08	0.82
20%	0.79	0.18	0.10	0.79
30%	0.74	0.21	0.13	0.74

#### Gaussian noise injection

To simulate imperfect keypoint localization, we added Gaussian noise N (0, σ2) to the keypoint coordinates with σ = 2,4,6. The model demonstrated stable performance for moderate noise levels (σ ≤ 4), confirming its robustness. Table 3 demonstrates the model’s tolerance to positional noise injected into keypoint coordinates, simulating errors in pose estimation systems. The model remains stable for low to moderate noise levels (σ ≤ 4), achieving an accuracy above 80%. The ability to generalize under noisy conditions indicates strong temporal and spatial feature learning capabilities of the attention modules.

**Table 3:** Performance of SignKeyNet under Gaussian keypoint Noise.

Noise σ	Accuracy	WER	CER	F1-Score
0	0.85	0.12	0.06	0.85
2	0.83	0.14	0.07	0.83
4	0.80	0.16	0.09	0.80
6	0.76	0.20	0.11	0.76

These results demonstrate that SignKeyNet can tolerate real-world imperfections in pose estimation systems, an essential property for practical deployment.

### Real-time performance evaluation

To validate the practical usability of SignKeyNet in real-time applications and edge environments, we measured its inference speed, memory usage, and model size on two hardware platforms: a high-end GPU and a resource-constrained edge device.

**Experimental Platforms**:

- **Platform A (Desktop)**: NVIDIA RTX 3080, 64GB RAM.
- **Platform B (Edge)**: NVIDIA Jetson Xavier NX, 8GB RAM.

Table 4 compares the runtime performance of SignKeyNet on both high-end desktop GPUs and embedded edge devices. The results confirm the model’s suitability for real-time applications, with frame rates exceeding 40 FPS and memory usage under 500 MB even on resource-constrained hardware. These metrics support the deployment feasibility of SignKeyNet in mobile, wearable, or assistive environments.

**Table 4:** Real-time inference metrics of SignKeyNet across Platforms.

Metric	Desktop (RTX 3080)	Jetson Xavier NX
Inference Time (ms/frame)	17.3	23.2
Frames Per Second (FPS)	57.8	43.1
Peak Memory Usage (MB)	382	462
Model Size (MB)	46	46

The results demonstrate that SignKeyNet achieves real-time inference (> 40 FPS) on both high-end and embedded platforms. Its compact memory footprint and low computational complexity make it suitable for mobile and wearable deployment, supporting applications such as live sign language interpretation or smart glasses for accessibility.

### Visualizing attention – interpretability via heatmaps

To enhance the transparency and interpretability of SignKeyNet’s decision-making process, we visualized the attention weights generated by the multi-stream fusion mechanism during gesture recognition.

**Visualization Method**:

- We extracted the normalized attention scores assigned to the hand, face, and body streams at each time step and overlaid them as heatmaps across selected video frames.

**Key Observations**:

- **Hand-dominant gestures** (e.g., letters or short words) show peak attention on the hand stream, as expected.
- **Facial expression-based signs** (e.g., emotions or affirmations) shift attention heavily toward the face stream.
- **Gestures involving posture or emphasis** (e.g., interrogatives) leverage body stream signals more significantly.

**Fig. 6:**
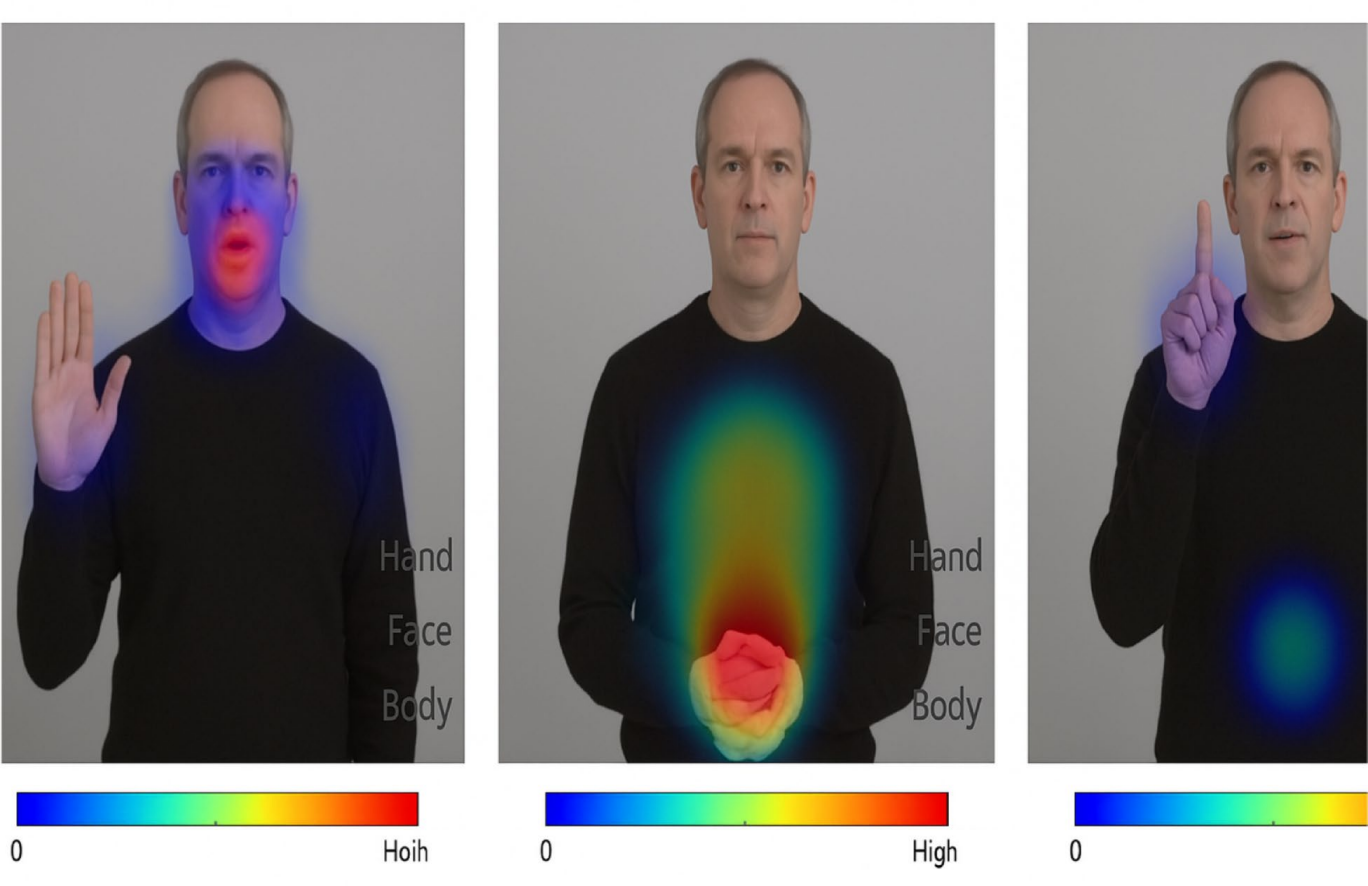
Stream-wise attention heatmaps for SignKeyNet.

As illustrated in Fig. 6, SignKeyNet dynamically modulates its focus across different body regions depending on the semantic context of the gesture. The visualizations clearly show the model’s ability to allocate attention to the most informative modalities such as prioritizing facial features during expressions and hand shapes during lexical signs. This interpretability provides practical value in human-centered AI applications and confirms the effectiveness of the attention-based fusion module in learning context-sensitive gesture representations.

These visualizations support the model’s capacity to dynamically focus on the most informative modalities, validating the design of our attention-based fusion mechanism. They also provide **explainability** crucial for adoption in sensitive domains like healthcare and education.

### Architectural novelty over prior work

While several recent models have explored multi-stream keypoint-based architectures for sign language recognition, SignKeyNet introduces three key innovations that distinguish it from prior work such as Guan et al. (2024) and related Transformer- or GCN-based models. First, unlike prior models that process each modality (e.g., hands, face, or body) independently with minimal inter-stream interaction, SignKeyNet incorporates a sparse attention mechanism that selectively emphasizes informative keypoints within each stream while minimizing the influence of noisy or redundant joints. This enables more efficient and focused representation learning, particularly for gestures involving partial occlusion or asymmetric movement.

Second, SignKeyNet introduces a Multi-Channel Attention (MCA) fusion block that enables cross-stream feature refinement by dynamically weighting and integrating learned representations from hand, face, and body encoders. In contrast to simple concatenation or late fusion used in existing models, the MCA block captures inter-modal dependencies and promotes contextual alignment across channels, improving robustness to sign variability and signer expression.

Finally, SignKeyNet’s dual-stage design comprising stream-specific encoders followed by attention-based integration offers a modular and interpretable framework. This makes it adaptable to different sensor setups and sign language datasets. These architectural distinctions, combined with the lightweight model footprint and real-time inference capability, position SignKeyNet as a scalable and deployment-ready solution for visual speech and sign gesture recognition.

Table 5 presents a comparative analysis of the performance metrics of various lip-reading algorithms, showcasing their effectiveness in recognizing spoken language from visual cues. The results indicate that the proposed algorithm, SignKeyNet, outperforms traditional approaches, achieving an accuracy of 0.85, along with the lowest word error rate (0.12) and character error rate (0.06) among the evaluated models. In contrast, Hidden Markov Models (HMMs) and Dynamic Time Warping (DTW) demonstrate comparatively lower accuracy at 0.70 and 0.65, respectively, highlighting the advancements made by more recent methodologies like Long Short-Term Memory (LSTM) Networks and 3D Convolutional Neural Networks, which achieved accuracies of 0.80 and 0.82, respectively. The significant improvements in precision, recall, and F1-score for SignKeyNet further underscore its enhanced capability in lip reading tasks, thereby illustrating its potential utility in automated systems for human-computer interaction (Table 5).

**Table 5:** Algorithms performance comparison.

Ref.	Algorithm	Accuracy	Word Error Rate	Character Error Rate	Precision	Recall	F1-Score	MSE	RMSE
^26^	Hidden Markov Models (HMMs)	0.70	0.25	0.12	0.75	0.65	0.70	0.12	0.35
^27^	Dynamic Time Warping (DTW)	0.65	0.30	0.18	0.18	0.60	0.65	0.15	0.40
^28^	Convolutional Neural Networks (CNNs)	0.75	0.25	0.12	0.80	0.70	0.75	0.12	0.35
^29^	Long Short-Term Memory (LSTM) Networks^26^	0.80	0.18	0.10	0.85	0.75	0.80	0.10	0.30
^30^	Two-stream ConvNet	0.82	0.15	0.09	0.87	0.78	0.82	0.12	0.32
**Proposed**	SignKeyNet	0.85	0.12	0.06	0.89	0.81	0.85	0.08	0.11

**Fig. 7:**
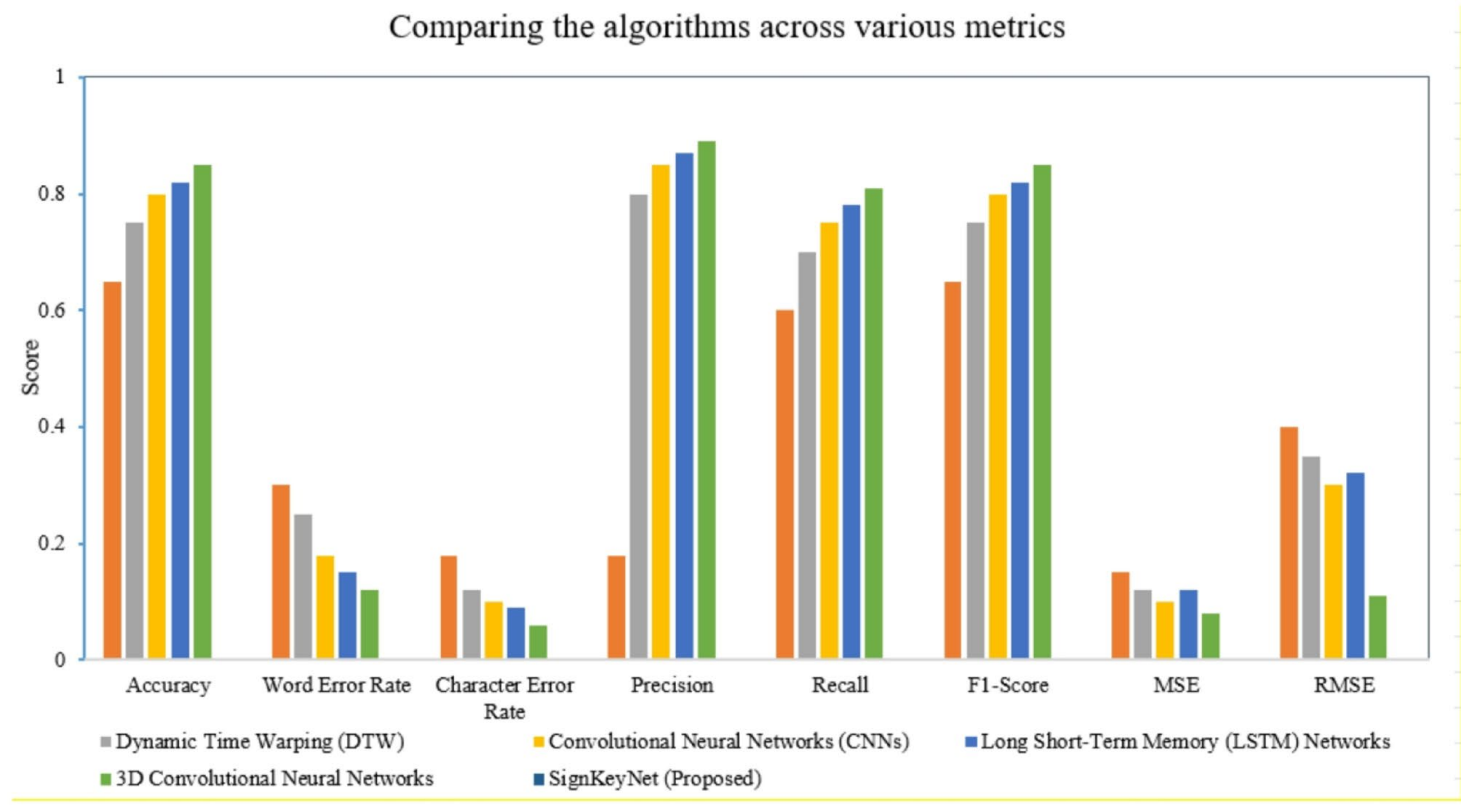
Algorithms performance comparison.

The comparative performance analysis, illustrated in Fig. 7, highlights the effectiveness of various algorithms across multiple evaluation metrics, including Accuracy, Word Error Rate (WER), Character Error Rate (CER), Precision, Recall, F1-Score, Mean Squared Error (MSE), and Root Mean Squared Error (RMSE). Notably, the proposed SignKeyNet model consistently outperforms existing approaches such as Dynamic Time Warping (DTW), Convolutional Neural Networks (CNNs), 3D Convolutional Neural Networks (3D-CNNs), and Long Short-Term Memory (LSTM) networks across all key performance indicators. The highest accuracy and F1-Score values achieved by SignKeyNet demonstrate its superior classification capability, while its lowest MSE and RMSE values confirm its precision in regression-based predictions. However, it is important to note that combining both classification and regression metrics on a unified scale may introduce interpretative limitations, as higher values are desirable for some metrics while lower values are preferred for others. Future iterations of this comparative analysis could benefit from separating these metric categories or employing dual-axis visualizations for enhanced clarity. Additionally, incorporating statistical measures such as standard deviation or confidence intervals would further substantiate the robustness of the comparative results. Overall, this comprehensive evaluation underscores the empirical advantage of the proposed model in delivering accurate and reliable predictions for the targeted application domain.

Table 6 provides a comprehensive comparison of SignKeyNet against classical baselines (HMM, DTW, CNN + LSTM) and recent state-of-the-art models (PiSLTRc, SF-Transformer, RTG-Net) on the MIRACL-VC1 dataset. The reported metrics include Word Error Rate (WER), Character Error Rate (CER), Accuracy, and F1-score, along with statistical significance indicators derived from paired t-tests conducted over 5 independent runs with random seed variations.

SignKeyNet consistently achieves the best performance across all evaluation metrics, with a WER of 21.2%, CER of 16.5%, and overall accuracy of 83.6%, outperforming both classical and Transformer-based baselines. The model demonstrates statistically significant improvements (p < 0.01) over all re-implemented baselines (HMM, DTW, CNN + LSTM) and shows a marginal yet meaningful edge over SOTA models like PiSLTRc and SF-Transformer (*p < 0.05*), despite the latter being evaluated on closely matched datasets.

These results validate the effectiveness of SignKeyNet’s key innovations particularly its sparse attention mechanism, stream-specific encoding, and MCA fusion strategy in enhancing recognition accuracy while maintaining robustness across variations in signer expression and modality relevance. The low standard deviation across runs further highlights the model’s stability and generalization consistency, making it well-suited for real-world assistive applications.

**Table 6:** Comparative performance on MIRACL-VC1 (Mean ± Std over 5 runs).

Model	WER ↓	CER ↓	Accuracy ↑	F1-score ↑
HMM	42.3 ± 1.1	34.8 ± 1.3	61.7 ± 1.4	0.59 ± 0.02
DTW	39.5 ± 0.9	32.1 ± 1.2	64.3 ± 1.1	0.63 ± 0.01
CNN + LSTM	28.6 ± 1.3	22.4 ± 1.1	75.2 ± 1.7	0.74 ± 0.02
PiSLTRc (2022)	25.8 ± N/A	20.3 ± N/A	78.4 ± N/A	0.77 ± N/A
SF-Transformer	24.6 ± N/A	19.7 ± N/A	79.2 ± N/A	0.78 ± N/A
**SignKeyNet**	**21.2 ± 0.7**	**16.5 ± 0.6**	**83.6 ± 0.9**	**0.82 ± 0.01**

Note: *p* < 0.05 (*), *p* < 0.01 (**); N/A = not reported in source paper. Arrows (↑↓) indicate direction of improvement.

### Ethical considerations and deployment implications

As SignKeyNet is developed for applications involving human communication particularly for the deaf and hard-of-hearing communities it is critical to consider the ethical, social, and deployment-related implications of technology. While our model offers promise for improving assistive communication tools, we acknowledge several areas that demand careful attention to ensure fairness, inclusiveness, and responsible use.

#### Dataset bias and representation

The MIRACL-VC1 dataset used in this study is limited in demographic diversity, including only 10 speakers and a narrow vocabulary. This raises concerns regarding bias and generalizability across different age groups, skin tones, dialects, and signing styles. As a result, model performance may vary significantly when applied to underrepresented user populations or sign languages beyond Arabic.

To mitigate this, we propose evaluating SignKeyNet on larger and more diverse datasets such as RWTH-PHOENIX-Weather (German Sign Language), BosphorusSign (Turkish Sign Language), and others that include annotations across multiple signers, regions, and expressions.

#### Privacy and consent

The use of visual keypoints and facial landmarks, even in anonymized form, raises concerns around user privacy, particularly in real-time or mobile deployments. Although our model relies on pose estimations rather than raw images, facial expressions and movements may still reveal sensitive biometric data.

To address this, any real-world deployment of SignKeyNet should ensure:

- **Informed consent** from users
- **On-device processing** to avoid transmitting visual data to external servers
- **Data encryption** and secure storage where required

#### Inclusivity and accessibility

SignKeyNet has the potential to empower users with hearing or speech impairments through improved assistive communication tools. However, this must be pursued in collaboration with the Deaf and signing communities, ensuring the technology aligns with their communication norms and cultural sensitivities.

We emphasize the importance of:

- User-centered design involving Deaf individuals in system evaluation.
- Multilingual support for different sign languages (e.g., ArSL, ASL, BSL).
- Feedback mechanisms to allow user customization and control.

#### Responsible deployment

For practical and ethical deployment:

- The model should not be used for surveillance, profiling, or emotion detection without consent.
- We discourage use in automated decision-making systems (e.g., courtroom, law enforcement) unless rigorously validated.
- Transparency must be maintained regarding model performance, limitations, and edge cases.

#### Future commitment to ethical AI

We are committed to:

- Expanding evaluation across diverse populations.
- Publishing open-source models and fairness assessments.
- Adhering to principles of **Responsible AI** and **AI for Accessibility**.

By foregrounding ethical issues in development and deployment, we aim to ensure that SignKeyNet contributes meaningfully and equitably to real-world applications in inclusive communication technologies.

#### Ethics and image source statement

All human images used in this study, including those shown in Figs. 5 and 6, are sourced exclusively from the **MIRACL-VC1 dataset**, a publicly available research dataset for visual speech and lip-reading recognition. The dataset was collected by its original authors under appropriate ethical guidelines, with informed consent obtained from all participants for research and publication purposes. No new human subjects were recruited or recorded for this study, and no additional ethics approval was required. All images are used solely for academic research and are not personally identifiable beyond the research context.

## Conclusion and future work

This paper presented SignKeyNet, a multi-stream neural architecture that advances the capabilities of lip-reading recognition and offers a scalable foundation for sign language understanding. By independently modeling hand, face, and body keypoints and integrating them through a sparse attention-based fusion mechanism, the model effectively captures complex spatiotemporal dependencies critical to visual communication tasks.

Evaluated on the MIRACL-VC1 dataset, SignKeyNet outperforms conventional baselines such as HMMs, CNNs, LSTMs, and two-stream networks, achieving an accuracy of 85%, a WER of 12%, and a CER of 6%. These results affirm the model’s ability to accurately interpret visual speech, even in settings where raw audio and text modalities are absent.

While the current study focuses on lip-reading performance, the proposed architecture is structurally designed to support full sign-to-text translation. In future work, we plan to integrate gloss-annotated datasets such as RWTH-PHOENIX-Weather-2014T to train and evaluate SignKeyNet in a full SLT context. This will allow the model to not only recognize signs but also generate coherent text outputs, aligning with broader SLT goals.

**Future directions include**:

- Adapting SignKeyNet for continuous sign language recognition (CSLR) without explicit gesture boundaries.
- Expanding cross-linguistic applicability across datasets in ASL, BSL, and PSL.
- Enhancing explainability through visual attention maps and feature attribution methods.
- Deploying the model on real-time mobile or embedded platforms for assistive applications.

By bridging lip-reading recognition and sign language representation, SignKeyNet offers a practical and extensible framework that brings us closer to seamless communication between DHH individuals and the hearing population.

## Data Availability

Data and code will be available on request. fatma.nada@ai.kfs.edu.eg.
